# Spondylodiscitis in a tuberculosis-endemic setting in Somalia: epidemiological, clinical, radiological, and outcome findings from a retrospective study

**DOI:** 10.3389/ftubr.2026.1971442

**Published:** 2026-10-09

**Authors:** Hussein Salad Yusuf, Abdirisak Ali Abukar, Mohamed Said Adan, Abdiaziz Siad Mohamed, Zakariye Yusuf Mohamed, Mustaf Abdi Ulusow, Mohamed Osman Dahir Alasow, Abdiwahid Ahmed Ibrahim, Naimo Awil Dahir

**Affiliations:** 1 Department of Neurosurgery, Mogadishu Somali Turkey Recep Tayyip Erdogan Training and Research Hospital, Mogadishu, Somalia; 2 Department of Radiology, Mogadishu Somali Turkey Recep Tayyip Erdogan Training and Research Hospital, Mogadishu, Somalia; 3 Department of Neurology, Mogadishu Somali Turkey Recep Tayyip Erdogan Training and Research Hospital, Mogadishu, Somalia; 4 Department of Pulmonology, Mogadishu Somali Turkey Recep Tayyip Erdogan Training and Research Hospital, Mogadishu, Somalia

**Keywords:** clinical characteristics, epidemiology, radiology, Somalia, spinal tuberculosis, spondylodiscitis, treatment outcome

## Abstract

**Background:**

Spondylodiscitis is an important cause of spinal morbidity in tuberculosis (TB)-endemic settings, where limited microbiological capacity may complicate differentiation of tuberculous from non-tuberculous disease. Data describing the clinical spectrum and outcomes of spondylodiscitis in Somalia are limited.

**Objective:**

To describe the demographic, clinical, radiological, microbiological, treatment, and outcome characteristics of patients with spondylodiscitis and to explore factors associated with clinical outcome.

**Methods:**

We conducted a retrospective observational study of 121 eligible patients diagnosed with spinal infection at Mogadishu Somali Turkey Recep Tayyip Erdoğan Training and Research Hospital between 1 August 2023 and 31 July 2024. Demographic, clinical, radiological, microbiological, treatment, and outcome data were extracted from routine records. Associations were assessed using chi-square or Fisher's exact tests for categorical variables and Mann–Whitney U or independent-samples t-tests as appropriate for continuous variables. Outcome comparisons were restricted to patients with documented follow-up; multivariable logistic regression was not retained because only 19 unfavorable outcomes were available and outcome ascertainment was incomplete.

**Results:**

The mean age was 48.8 ± 18.1 years and 53.7% were male. Back pain (92.6%) and radicular pain (82.6%) were the most frequent presenting symptoms. Neurological deficit was documented in 46.3% and spinal deformity in 38.0%. Lumbar involvement was most common (48.8%), followed by thoracic involvement (25.6%); anterior-column involvement occurred in 97.5%. Epidural abscess was identified in 69.4% and is reported here as a radiological finding. Microbiological or histopathological testing was performed in 46 patients; 30 were TB-positive, corresponding to 65.2% positivity among tested patients and 24.8% confirmed TB among the total cohort. Eighty-five patients (70.2%) received empirical anti-TB treatment. Among 65 patients with documented follow-up, 46 (70.8%) achieved favorable outcomes. In bivariate analysis, TB exposure history was associated with outcome (*p* = 0.003), whereas operative/procedural intervention was not significantly associated with outcome (Fisher's exact *p* = 0.399).

**Conclusion:**

Spondylodiscitis in this Somali tertiary-care cohort showed a substantial burden of neurological and radiological complications and a high proportion of empiric TB treatment. Because microbiological confirmation was limited and follow-up was incomplete, the observed confirmed TB proportion and favorable-outcome estimate should not be interpreted as population-level estimates. Prospective multicenter studies with systematic microbiological testing and complete follow-up are needed to clarify the etiological spectrum and prognostic factors.

## 1 Introduction

Spondylodiscitis, including vertebral osteomyelitis and associated spinal epidural infection, is an important cause of morbidity because delayed diagnosis can result in vertebral destruction, deformity, neurological impairment, and prolonged disability. In tuberculosis-endemic settings, spinal tuberculosis (STB) is an important cause of spinal infection and may present with persistent back pain, deformity, constitutional symptoms, or neurological compromise (1–4).

STB is usually acquired through hematogenous dissemination of *Mycobacterium tuberculosis* from a primary focus, most commonly the lungs; gastrointestinal or other extrapulmonary sources may also contribute (5, 6). The epidemiology of TB varies geographically, and the burden is strongly influenced by socioeconomic conditions, access to healthcare, diagnostic capacity, and delays in treatment (7–10).

The clinical presentation of spinal infection is frequently nonspecific. Persistent back pain is common, whereas fever, weight loss, and night sweats are variably present. Neurological deficits may develop with epidural extension, vertebral destruction, deformity, or compression (3, 4, 11). Because these findings overlap with pyogenic and other infectious causes, microbiological or histopathological confirmation is important whenever feasible.

In Somalia, clinicians must also consider brucellosis in the differential diagnosis of spinal TB, particularly in patients with compatible epidemiological exposure or systemic features. *Brucella* spondylodiscitis can closely resemble tuberculous or pyogenic spondylodiscitis clinically and radiologically, making appropriate laboratory evaluation important when available (12).

The management of spinal infection depends on the presumed etiology, neurological status, mechanical stability, abscess formation, and response to medical therapy. Anti-TB chemotherapy is the cornerstone of confirmed spinal TB, while surgery is generally reserved for selected patients with neurological compromise, instability, deformity, significant abscess formation, or failure of medical treatment (13–17).

Despite the clinical importance of spinal infection in TB-endemic settings, published data from Somalia describing the spectrum of spondylodiscitis, the burden of TB, and outcome predictors are limited. Therefore, this study evaluated 121 patients with spondylodiscitis treated at a tertiary referral hospital in Mogadishu. The objectives were to estimate the proportion of confirmed TB among the overall cohort and among tested patients, describe demographic, clinical and radiological characteristics, summarize microbiological and treatment findings, and identify factors associated with clinical outcome.

## 2 Methods

### 2.1 Study design and setting

This retrospective observational study reviewed medical records of patients diagnosed with spinal infection at Mogadishu Somali Turkey Recep Tayyip Erdoğan Training and Research Hospital, a tertiary referral and teaching hospital in Mogadishu, Somalia. The study period was 1 August 2023 to 31 July 2024. The study was designed as a record-based analysis of routinely collected clinical data; no prospective intervention or additional research-specific laboratory testing was performed.

### 2.2 Study population and eligibility

The study population comprised all eligible patients diagnosed with spinal infection during the study period. Eligible conditions included spondylodiscitis, vertebral osteomyelitis, and spinal epidural abscess with radiological evidence on MRI, CT, or x-ray. Microbiological or histopathological confirmation was included when available. Records were excluded when they were incomplete, duplicated, or lacked radiological confirmation. Total-enumeration sampling was used.

Two analytical populations were prespecified. The full cohort (*N* = 121) was used for demographic, clinical, radiological, microbiological, treatment, and TB-burden analyses. Outcome analyses were restricted to the 65 patients with documented follow-up. Analyses of TB confirmation status were restricted to the 46 patients who underwent microbiological or histopathological testing.

### 2.3 Data collection and variables

Data were extracted from medical records, laboratory databases, radiology reports, and operative notes. Sociodemographic variables included age, sex, occupation, marital status, residence, and reported economic status. Clinical history included pulmonary TB, extrapulmonary TB, TB exposure, trauma, and chronic comorbidity. Clinical presentation included back pain, radicular pain, bowel/bladder dysfunction, constitutional symptoms, muscle power, and spinal deformity.

Radiological variables included vertebral level, anterior-, middle-, and posterior-column involvement, epidural abscess, and associated disc herniation. Epidural abscess and disc herniation were treated as radiological findings rather than clinical characteristics. Treatment variables included operative/procedural status, type of procedure, anti-TB treatment, and microbiological/pathological findings. Percutaneous biopsy was classified as a diagnostic procedure rather than a therapeutic operation.

Neurological status was assessed using the ASIA Impairment Scale where documented; the Frankel scale was used for historical comparison. Clinical outcome was categorized as complete recovery, partial recovery, or no recovery. For bivariate outcome comparisons, favorable outcome was defined as complete or partial recovery, while unfavorable outcome was defined as no recovery. Radiological follow-up with MRI was used when available to assess compression and lesion resolution.

### 2.4 Operational definitions

TB-related spondylodiscitis was defined as spinal infection with microbiological evidence of M. tuberculosis, histopathological findings suggestive of TB, or strong clinical and radiological suspicion supported by response to anti-TB treatment. Pyogenic infection was defined as spinal infection with a positive bacterial culture excluding M. tuberculosis. Fungal infection was defined by microbiological confirmation of a fungal organism. Confirmed TB prevalence was calculated as confirmed TB cases divided by all 121 patients; positivity among tested patients was calculated as confirmed TB cases divided by the 46 patients who underwent testing. Empirical or presumptive TB burden was represented by the proportion of the full cohort that received anti-TB treatment, regardless of microbiological confirmation.

Age was analyzed as a continuous variable and in four categories (18–30, 31–45, 46–60, and >60 years). Neurological deficit was defined as any documented reduction in muscle power or other neurological impairment attributable to spinal disease. Economic status was categorized as low, middle, or high according to the socioeconomic classification recorded in the medical record. TB exposure and trauma were defined according to documented history in the clinical record.

### 2.5 Laboratory and radiological assessment

Routine laboratory investigations included C-reactive protein, erythrocyte sedimentation rate, and microbiological cultures when clinically indicated. Tissue or other diagnostic specimens were evaluated using the laboratory methods available during routine care. Radiological assessment was performed by hospital radiologists using MRI, CT, and/or x-ray as clinically indicated.

### 2.6 Ethics

Ethical approval was obtained from the Ethics Committee of Mogadishu Somalia–Turkey Recep Tayyip Erdoğan Training and Research Hospital (Approval No. 1294). The study used anonymized routinely collected hospital data, and the requirement for informed consent was waived by the Ethics Committee. Patient confidentiality was maintained throughout the study. No additional investigations were performed for research purposes.

### 2.7 Statistical analysis

Data were analyzed using IBM SPSS Statistics version 23.0. Continuous variables were summarized using mean ± standard deviation for approximately normally distributed data and median with interquartile range for skewed data; categorical variables were summarized using counts and percentages. Distribution of age was assessed using the Shapiro–Wilk test. Categorical variables were compared using the chi-square test or Fisher's exact test when expected cell counts were small. Continuous variables were compared using the independent-samples t-test or Mann–Whitney U test, as appropriate. Spearman rank correlation was used for selected associations. Because only 19 unfavorable outcomes were available among the 65 patients with documented follow-up, and outcome data were unavailable for 56 of the 121 patients, multivariable logistic regression was not retained in the final analysis. Outcome associations are therefore presented as unadjusted bivariate comparisons, with emphasis on effect direction, sample size, and the limitations of incomplete follow-up.

## 3 Results

### 3.1 Demographic and socioeconomic characteristics

A total of 121 eligible patients were included. Mean age was 48.8 ± 18.1 years (median 50; IQR 33–62; range 17–91), with a non-normal distribution (Shapiro–Wilk W = 0.976, *p* = 0.031). The largest age group was 46–60 years (31.4%, *n* = 38), followed by >60 years (28.1%, *n* = 34). Age-group frequencies are presented in (Table 1). Males comprised 53.7% (*n* = 65), (Figure 1). Detailed demographic characteristics are presented in Table 1.

**Figure 1:**
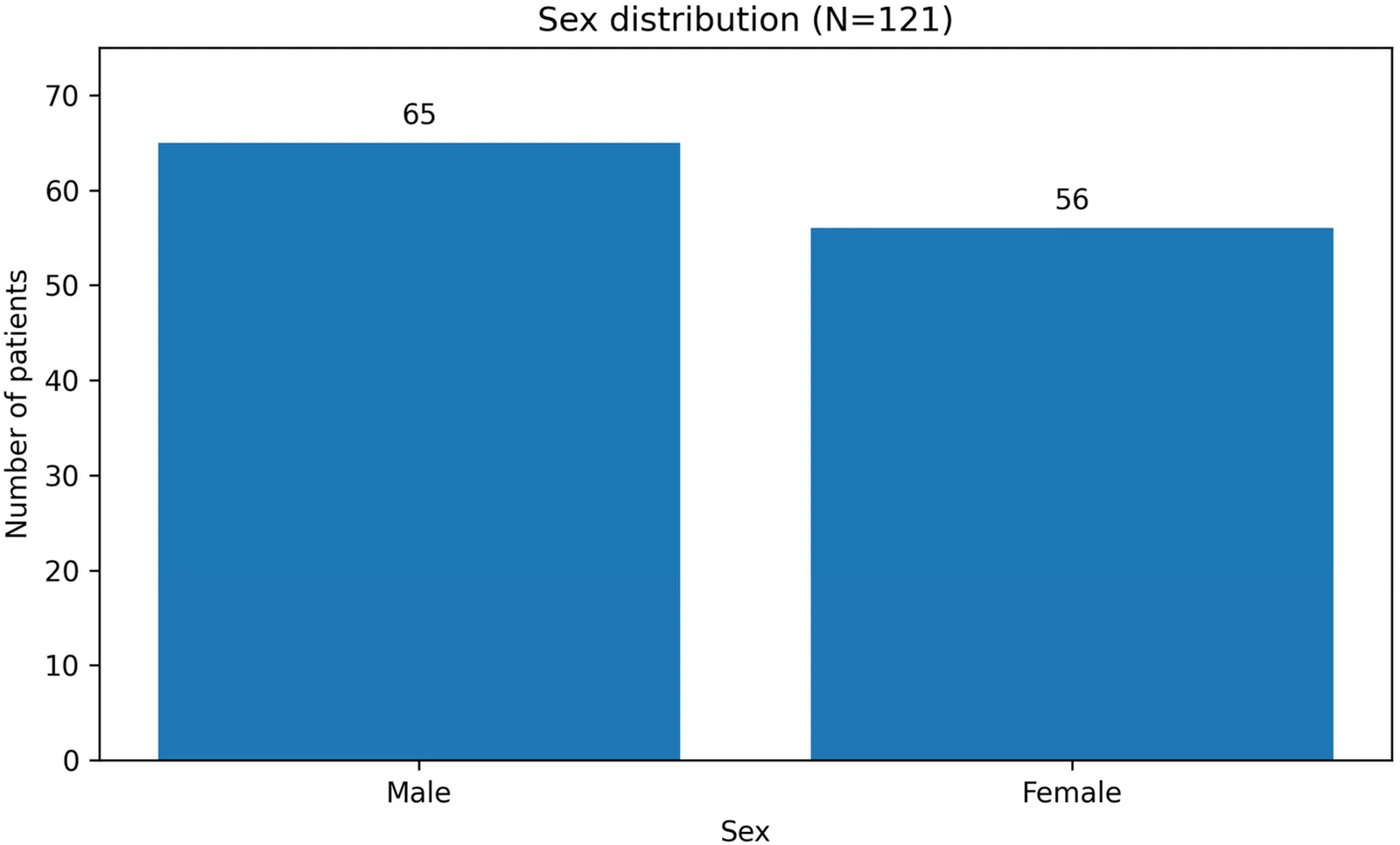
Bar chart showing sex distribution in the studied population (*N* = 121).

**Table 1:** Demographic and socioeconomic characteristics of patients with spondylodiscitis (*N* = 121).

Variable	Category	n	%
Age (years)	Mean ± SD	48.8 ± 18.1	
	Median (IQR)	50 (33–62)	
	Range	17–91	
Age group	18–30	22	18.2
	31–45	27	22.3
	46–60	38	31.4
	>60	34	28.1
Sex	Male	65	53.7
	Female	56	46.3
Occupation	Housewife	33	27.3
	Unemployed	22	18.2
	Retired	21	17.4
	Laborer	12	9.9
	Driver	8	6.6
	Student	8	6.6
	Shopkeeper	5	4.1
	Farmer	5	4.1
	Teacher	4	3.3
	Soldier	3	2.5
Marital status	Married	93	76.9
	Single	19	15.7
	Widow	5	4.1
	Divorced	4	3.3
Residence	Urban	93	76.9
	Rural	28	23.1
Economic status	Low	55	45.5
	Middle	61	50.4
	High	5	4.1

### 3.2 Clinical characteristics and TB-related history

A history of pulmonary TB was documented in 13 patients (10.7%) and extrapulmonary TB in one patient (0.8%). TB exposure was reported in 23.1% (*n* = 28), while 45.5% (*n* = 55) had a history of trauma. Chronic comorbidity was documented in 12.4%; diabetes mellitus was the most common comorbidity (6.6%, *n* = 8). Back pain (92.6%, *n* = 112) and radicular pain (82.6%, *n* = 100) were the predominant symptoms. Constitutional symptoms were present in 50.4% (*n* = 61), bowel/bladder dysfunction in 10.7% (*n* = 13), neurological deficit in 46.3% (*n* = 56), and spinal deformity in 38.0% (*n* = 46). Among the 56 patients with documented neurological deficit, muscle-power grades were grade 4 in 21 (37.5%), grade 3 in 15 (26.8%), grade 2 in 13 (23.2%), grade 1 in 1 (1.8%), and grade 0 in 6 (10.7%). Specific ASIA/Frankel categories and patient-level linkage between pre-operative neurological grade and outcome were not consistently available in the retrospective records; therefore, neurological recovery could not be mapped reliably to ASIA/Frankel grade. These findings are summarized in Table 2.

**Table 2:** Clinical characteristics and TB history (*N* = 121).

Variable	n	%
Pulmonary TB	13	10.7
Extrapulmonary TB (brain)	1	0.8
History of TB exposure	28	23.1
History of trauma	55	45.5
Chronic disease		
*Diabetes mellitus*	8	6.6
*HBsAg + ve*	4	3.3
*Chronic kidney failure*	2	1.7
*HCV + ve*	1	0.8
Back pain	112	92.6
Radicular pain	100	82.6
Bowel and bladder dysfunction	13	10.7
Constitutional symptoms	61	50.4
Neurological deficit (any grade)	56	46.3
Muscle power grades*	56	46.3
*Grade 4*	21	17.4
*Grade 3*	15	12.4
*Grade 2*	13	10.7
*Grade 1*	1	0.8
*Grade 0*	6	5.0
Spinal deformity	46	38.0
Epidural abscess	84	69.4
Associated disc herniation	48	39.7
History of spine surgery	5	4.1

*Muscle-power grades are reported among the 56 patients with neurological deficit.

### 3.3 Radiological findings

The lumbar spine was the most frequently involved region (48.8%, *n* = 59), followed by the thoracic spine (25.6%, *n* = 31), (Figure 2). Anterior-column involvement was present in 97.5% (*n* = 118), middle-column involvement in 72.7% (*n* = 88), and posterior-column involvement in 21.5% (*n* = 26) (Figure 3). Epidural abscess was present in 69.4% (*n* = 84), and associated disc herniation in 39.7% (*n* = 48). Spinal involvement details are shown in Table 3.

**Figure 2:**
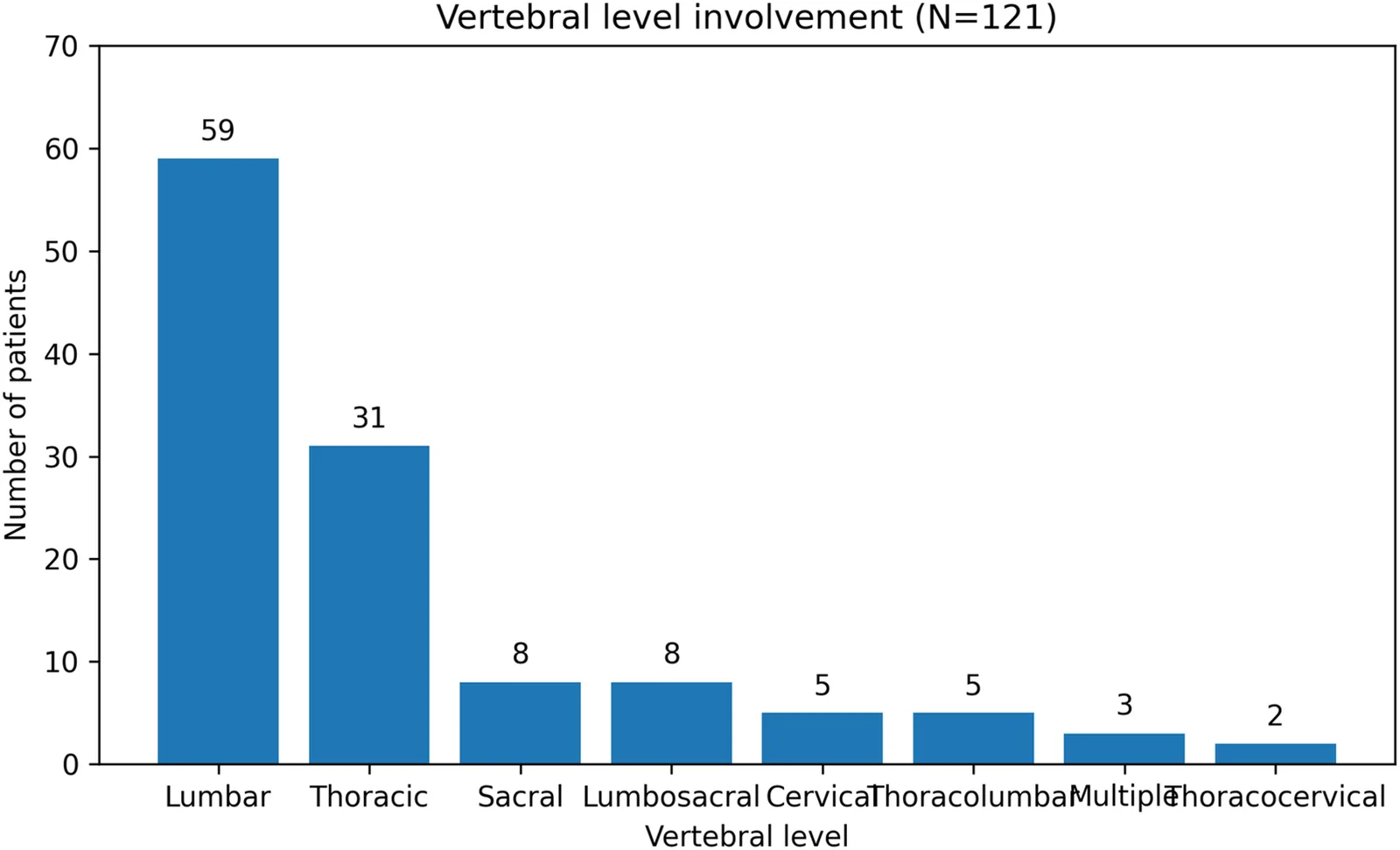
Bar chart showing vertebral level involvement (*N* = 121).

**Figure 3:**
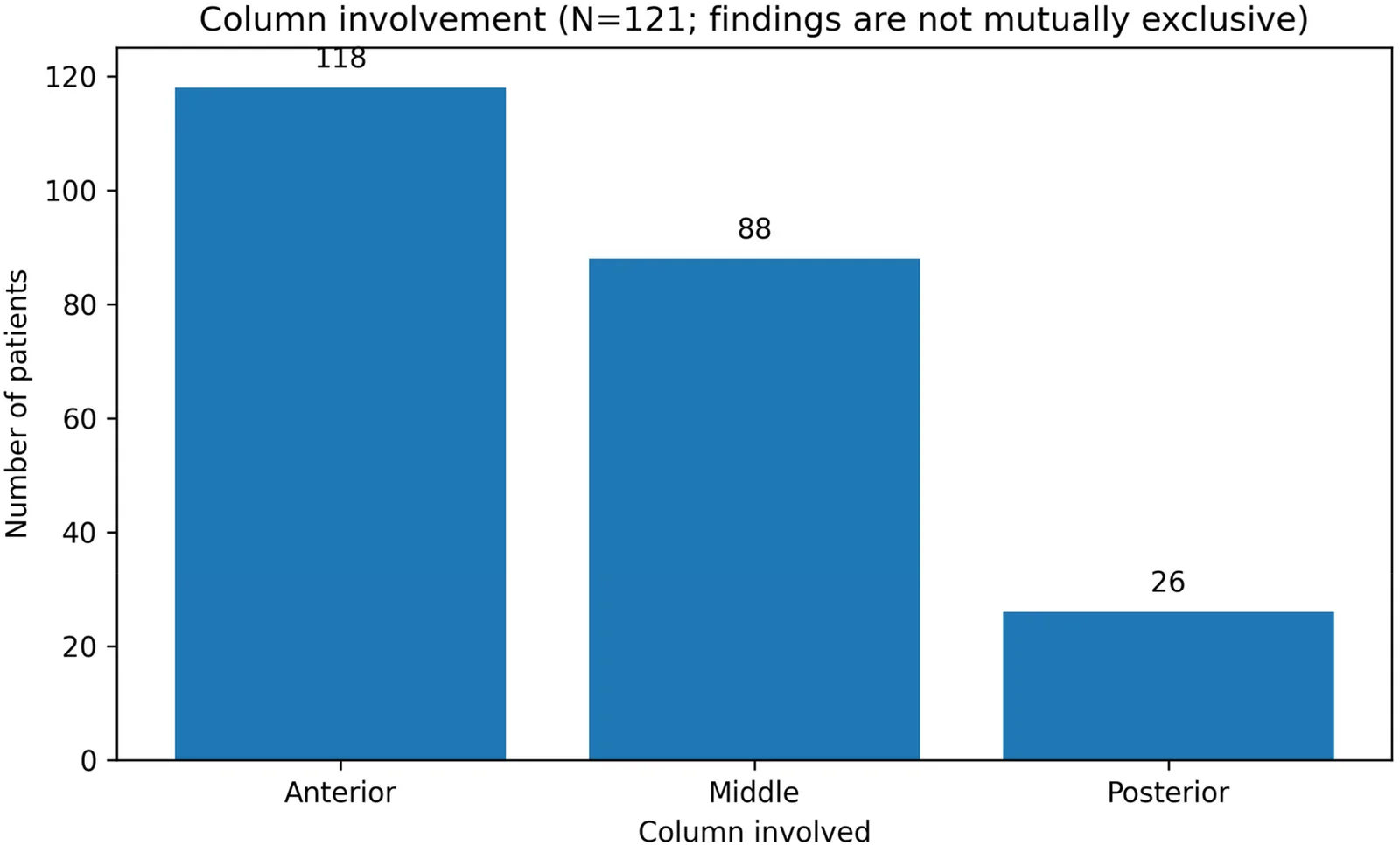
Bar chart showing anterior-, middle-, and posterior-column involvement. Findings are not mutually exclusive; *N* = 121 is the denominator for each finding.

**Table 3:** Spinal involvement and radiological findings (*N* = 121).

Variable	Category	n	%
Vertebral level	Lumbar	59	48.8
	Thoracic	31	25.6
	Sacral	8	6.6
	Lumbosacral	8	6.6
	Cervical	5	4.1
	Thoracolumbar	5	4.1
	Multiple	3	2.5
	Thoracocervical	2	1.7
Column involvement	Anterior	118	97.5
	Middle	88	72.7
	Posterior	26	21.5
Associated disc herniation	Yes	48	39.7
	No	73	60.3

Spinal tuberculosis (STB) can involve any level of the vertebral column, including the lumbar, thoracic, and cervical regions. Involvement may be limited to a single spinal segment or occur as multilevel disease. The distribution of vertebral involvement demonstrates the variable pattern of STB throughout the spine (Figure 4).

**Figure 4:**
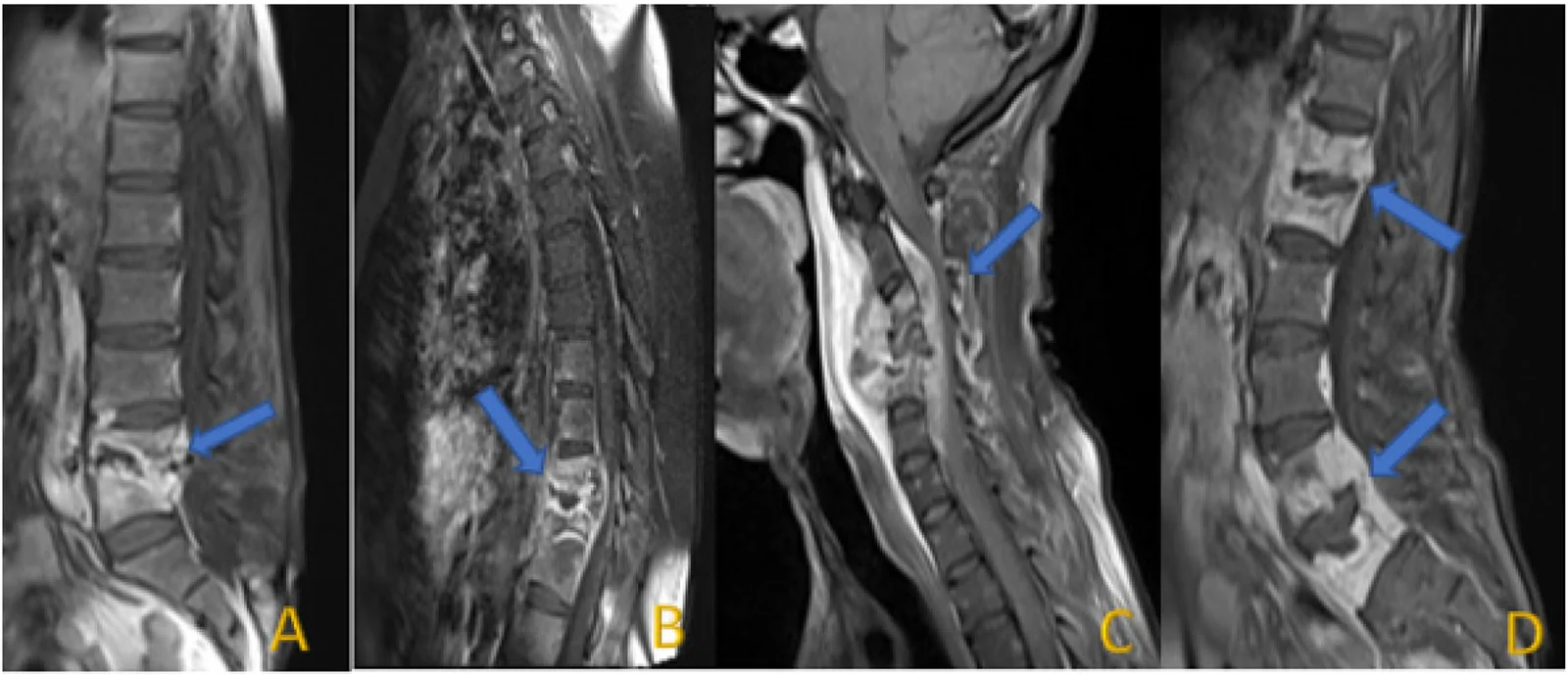
Representative sagittal MRI panels showing spondylodiscitis. **(A)** Lumbar lesion; **(B)** thoracic lesion; **(C)** cervical lesion; **(D)** multilevel lesions. Arrows indicate the principal abnormal vertebral/disc regions. MRI sequence details were not consistently available in the retrospective record.

**Figure 5:**
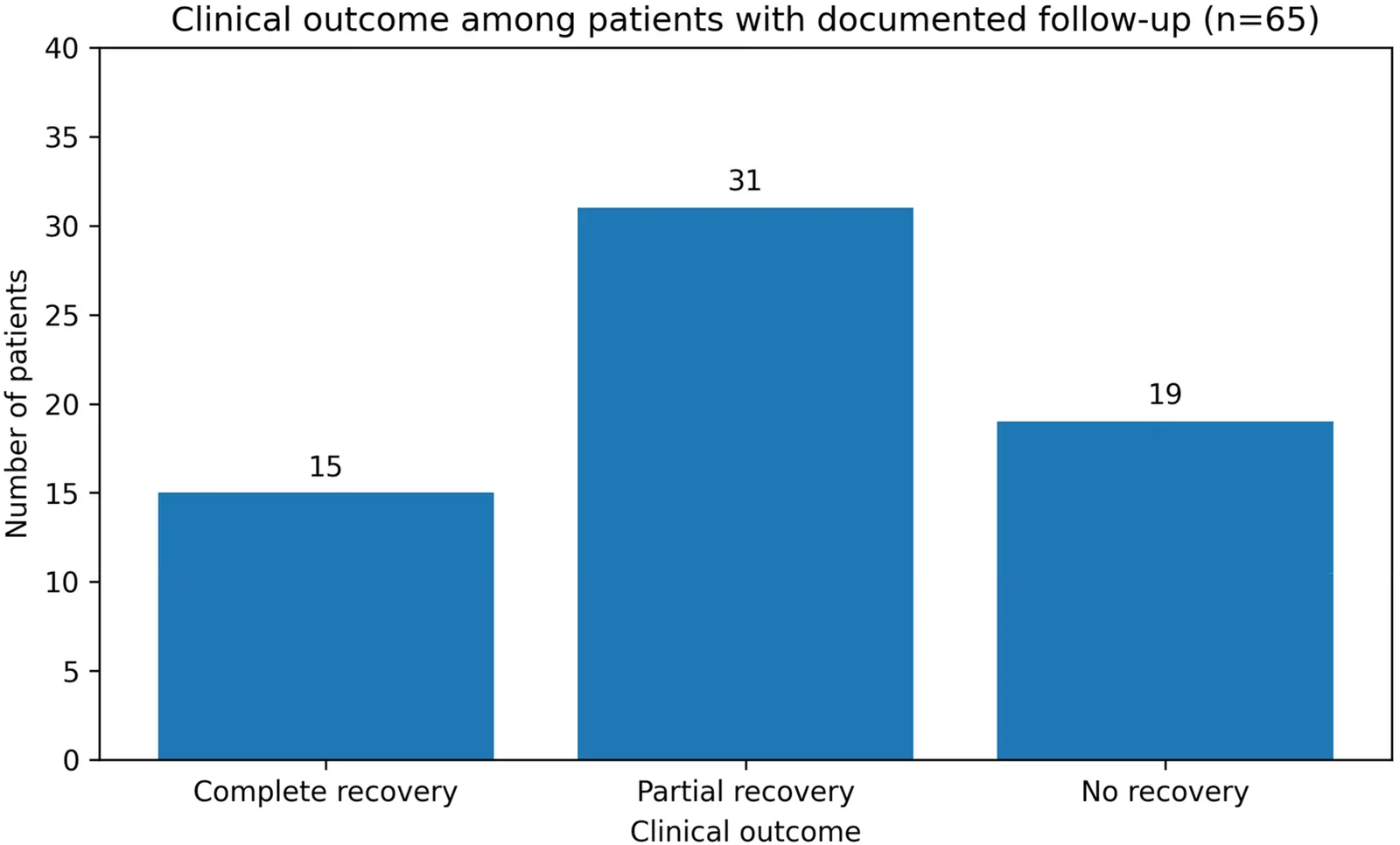
Bar chart showing clinical outcome among patients with documented follow-up (*n* = 65).

### 3.4 Microbiological findings, TB burden, and treatment

Microbiological or histopathological testing was performed in 46 patients (38.0%). Thirty of these patients were TB-positive, giving a positivity rate of 65.2% among tested patients and a confirmed TB proportion of 24.8% in the total cohort. Four patients had non-TB organisms identified: gram-positive cocci, Klebsiella spp., mucormycosis, and Staphylococcus epidermidis, one case each. Drug-susceptibility results were available for 29 of the 30 TB-positive patients; 25 (86.2%) had no documented resistance and 4 (13.8%) had monoresistance. No multidrug-resistant TB was identified among the tested confirmed TB cases. Drug-resistance categories were based on the 30 confirmed TB cases; susceptibility results were available for 29 patients.

Overall, 85 patients (70.2%) received anti-TB treatment. This proportion represents empirical/presumptive treatment exposure and should not be interpreted as microbiologically confirmed TB prevalence. Forty-six patients underwent an operative/procedural intervention; instrumentation with biopsy was the most common operative procedure. Percutaneous biopsy was considered a diagnostic procedure. The treatment and microbiological data are detailed in Table 4.

**Table 4:** Treatment, microbiological results, and prevalence (*N* = 121).

Variable	Category	n	%
Intervention category	Non-operated	75	62.0
	Operated	46	38.0
Specific intervention	Anti-TB Treatment Only	75	62.0
	Instrumentation + Biopsy	28	23.1
	Percutaneous Biopsy	14	11.6
	Laminectomy + Biopsy	3	2.5
	Retropharyngeal Abscess Drainage + Biopsy	1	0.8
Microb/pathology result	Not Done	75	62.0
	TB Positive	30	24.8
	No Result	12	9.9
	Gram Positive Cocci	1	0.8
	Klebsiella Spp	1	0.8
	Mucormycosis	1	0.8
	Staphylococcus Epidermidis	1	0.8
Drug resistance	No Result	92	76.0
	No Resistance	25	20.7
	Monoresistance	4	3.3
Anti-TB treatment	Yes	85	70.2
	No	36	29.8
Prevalence Estimates			
*TB-confirmed among tested (n = 46)*	30/46		65.2
*TB-confirmed among* *all patients (n = 121)*	30/121		24.8
*Presumptive + confirmed* *TB (anti-TB treated)*	85/121		70.2

### 3.5 Clinical outcomes

Documented follow-up was available for 65 patients (53.7%); 56 patients (46.3%) had no documented follow-up. Among the 65 patients with outcome data, 15 (23.1%) achieved complete recovery, 31 (47.7%) partial recovery, and 19 (29.2%) no recovery. Thus, 46/65 (70.8%) had favorable outcomes. Because outcome data were unavailable for nearly half of the cohort, this proportion applies only to patients with documented follow-up and should not be generalized to all 121 patients. A comparative analysis of baseline characteristics between patients with and without documented follow-up could not be reliably reconstructed from the available retrospective record extract because patient-level follow-up linkage was incomplete. The outcome distribution is presented in Table 5. Figure 5.

**Table 5:** Clinical outcomes in the overall cohort and Among patients With documented follow-up (*N* = 121).

Variable	Category	n	%
Clinical outcome (all patients)	Complete Recovery	15	12.4
	Partial Recovery	31	25.6
	No Recovery	19	15.7
	No Data	56	46.3
Clinical outcome (with follow-up, *n* = 65)	Complete Recovery	15	23.1
	Partial Recovery	31	47.7
	No Recovery	19	29.2
Outcome duration	Less Than 1 Year	33	27.3
	1 Year	7	5.8
	More Than 1 Year	25	20.7
	No Data	56	46.3

### 3.6 Outcome by intervention category

Among the 65 patients with documented outcomes, 43 had undergone surgery and 22 had not. Favorable outcome occurred in 32/43 (74.4%) operated patients and 14/22 (63.6%) non-operated patients. The three-category outcome distribution did not differ significantly (*χ*^2^ = 0.959, df = 2, *p* = 0.619). Dichotomized favorable versus unfavorable outcome was also not statistically significant in the unadjusted comparison (Fisher's exact *p* = 0.399). The detailed comparison is shown in Table 6.

**Table 6:** Clinical outcome by intervention category (*N* = 65).

(a)					
Intervention	Complete recovery n(%)	Partial recovery n(%)	No recovery n(%)	Total	*p*-value
Non-operated	4 (18.2)	10 (45.5)	8 (36.4)	22	0.619
Operated	11 (25.6)	21 (48.8)	11 (25.6)	43	

(b)				
Binary outcome	Favorable n(%)	Unfavorable n(%)	OR	*p*-value (Fisher)
Non-operated	14 (63.6)	8 (36.4)	0.60	0.399
Operated	32 (74.4)	11 (25.6)		

### 3.7 Bivariate analysis

Among the 65 patients with documented follow-up, TB exposure history was significantly associated with outcome (*p* = 0.003), and economic status also showed an association (*p* = 0.024). The apparent association with high economic status should be interpreted cautiously because only five patients were classified in this category. No other examined variable was statistically significant in the bivariate comparison. The full results are presented in Table 7.

**Table 7:** Bivariate analysis of factors associated with clinical outcome (*N* = 65).

Variable	Favorable n(%)	Unfavorable n(%)	*p*-value
Age (mean ± SD)	44.5 ± 18.0	47.3 ± 18.8	0.639
Sex (male)	28 (60.9)	12 (63.2)	1.000
Residence (urban)	40 (87.0)	13 (68.4)	0.156
Economic status			0.024*
*Low*	18 (69.2)	8 (30.8)	
*Middle*	27 (79.4)	7 (20.6)	
*High*	1 (20.0)	4 (80.0)	
Pulmonary TB	7 (100.0)	0 (0.0)	0.096
TB exposure history	4 (33.3)	8 (66.7)	0.003*
Trauma	23 (62.2)	14 (37.8)	0.139
Back pain	39 (67.2)	19 (32.8)	0.096
Radicular pain	40 (70.2)	17 (29.8)	1.000
Bowel/bladder dysfunction	6 (66.7)	3 (33.3)	0.714
Constitutional symptoms	23 (69.7)	10 (30.3)	1.000
Spinal deformity	18 (72.0)	7 (28.0)	1.000
Epidural abscess	36 (67.9)	17 (32.1)	0.484
Neurological deficit	27 (67.5)	13 (32.5)	0.651
Middle column	36 (66.7)	18 (33.3)	0.154
Posterior column	16 (72.7)	6 (27.3)	1.000
Disc herniation	18 (69.2)	8 (30.8)	1.000
Operative/procedural intervention	32 (74.4)	11 (25.6)	0.538
Anti-TB treatment	42 (71.2)	17 (28.8)	1.000

*Statistically significant at *p* < 0.05.

### 3.8 Correlation analysis

Because the variables analyzed in this section are binary or categorical, the associations are reported numerically in Table 8 using Spearman correlation coefficients. These coefficients should be interpreted as measures of association rather than evidence of causality.

**Table 8:** Correlation analysis Among key clinical variables.

Variable pair	r	*p*-value
Epidural abscess vs. middle column	0.762	<0.001**
Epidural abscess vs. neurological deficit	0.508	<0.001**
Neurological deficit vs. spinal deformity	0.366	<0.001**
Neurological deficit vs. operative/procedural intervention	0.366	<0.001**
Epidural abscess vs. spinal deformity	0.298	0.001**
TB exposure vs. constitutional symptoms	0.270	0.003**
TB exposure vs. outcome^a^	−0.392	0.001**
Trauma vs. outcome^a^	−0.218	0.082
Operative/procedural intervention vs. outcome^a^	0.112	0.374

a Outcome correlations among *n* = 65 (Favorable=1, Unfavorable=0); others among *N* = 121.

*p < 0.05, **p < 0.01.

### 3.9 Characteristics by TB confirmation status

Among the 46 tested patients, 30 (65.2%) were TB-positive and 16 (34.8%) were TB-negative or had another identified pathogen. TB-positive patients tended to be younger than the TB-negative/other group, but the difference was not statistically significant (37.8 ± 16.2 vs. 46.2 ± 17.4 years; *p* = 0.076). No statistically significant differences were observed in the other reported clinical or radiological characteristics. These results are summarized in Table 9.

**Table 9:** Characteristics by TB confirmation status.

Variable	TB Positive (*n* = 30)	TB Negative/Other (*n* = 16)	Test	*p*-value
Age (mean ± SD)	37.8 ± 16.2	46.2 ± 17.4	Mann–Whitney	0.076
Sex (male)	24 (80.0%)	9 (56.3%)	Chi-square	0.174
TB exposure history	9 (30.0%)	4 (25.0%)	Fisher	1.000
Back pain	28 (93.3%)	12 (75.0%)	Fisher	0.163
Radicular pain	29 (96.7%)	14 (87.5%)	Fisher	0.274
Constitutional symptoms	18 (60.0%)	8 (50.0%)	Chi-square	0.734
Spinal deformity	15 (50.0%)	7 (43.8%)	Chi-square	0.925
Epidural abscess	28 (93.3%)	14 (87.5%)	Fisher	0.602
Bowel/bladder dysfunction	5 (16.7%)	3 (18.8%)	Fisher	1.000
Neurological deficit	20 (66.7%)	10 (62.5%)	Chi-square	0.776

## 4 Discussion

This study describes the epidemiological, clinical, radiological, microbiological, treatment, and outcome characteristics of 121 patients with spondylodiscitis treated at a tertiary hospital in Mogadishu. The principal findings were the predominance of middle-aged patients, frequent back and radicular pain, a high frequency of neurological and radiological complications, a substantial proportion of confirmed TB among tested patients, extensive empirical anti-TB treatment, and incomplete follow-up. Importantly, the study included all spondylodiscitis cases rather than only confirmed TB, so the 24.8% confirmed TB proportion should be interpreted as a cohort-specific proportion rather than a population prevalence estimate.

### 4.1 Demographic profile

The mean age of 48.8 years was higher than the mean ages reported in some cohorts of spinal TB, including 28 years in a South African series and 36.9 years in another study, but was comparable to broader spinal infection populations and older cohorts (18–22). The male proportion in the present study was 53.7%, showing only a modest male predominance. Differences in age and sex distributions across studies may reflect differences in case definition, referral patterns, healthcare access, and whether cohorts included pyogenic as well as tuberculous infection.

Most patients were urban residents and were classified in low or middle socioeconomic groups. Socioeconomic disadvantage is a recognized risk context for TB, but the observed distribution should not be interpreted as causal because this study did not include a population-based control group (23–25). The apparent association between high economic status and unfavorable outcome in bivariate analysis is particularly unstable because only five patients were in the high-status category.

### 4.2 Clinical and radiological characteristics

Back pain was the dominant symptom (92.6%), consistent with previous studies of vertebral infection (26, 27). Radicular pain was also frequent, while constitutional symptoms occurred in only half of the cohort, emphasizing that absence of fever, weight loss, or night sweats does not exclude spinal infection. The 46.3% frequency of neurological deficit and 69.4% frequency of epidural abscess indicate a substantial burden of advanced or complicated disease at presentation. Among patients with neurological deficit, the recorded muscle-power grades ranged from grade 4 to grade 0. However, ASIA/Frankel grade and patient-level linkage between neurological grade and outcome were not consistently available, limiting more granular neurological outcome analysis.

Lumbar involvement was most frequent (48.8%), followed by thoracic involvement (25.6%). This pattern is broadly consistent with previous reports describing frequent lumbar and thoracolumbar involvement, although the distribution varies among populations (28, 29). Anterior-column involvement was present in nearly all patients, while middle-column involvement was also common.

The strong correlation between epidural abscess and middle-column involvement and the moderate correlation between epidural abscess and neurological deficit are clinically coherent, but correlation does not establish a temporal or causal relationship.

### 4.3 Microbiological confirmation and TB burden

Only 46 of 121 patients underwent microbiological or histopathological testing. Among these, 30 were TB-positive, giving a testing-positive proportion of 65.2%. In contrast, confirmed TB represented 24.8% of the entire cohort. These two denominators answer different questions and should not be conflated. The high proportion of empirical anti-TB treatment (70.2%) further illustrates the diagnostic constraints in this setting, but treatment exposure cannot be used as confirmation of TB.

The limited testing also means that the true etiological distribution remains uncertain. Some clinically presumed TB cases may have had pyogenic, fungal, brucellar, or other infections. Brucellosis is an important differential diagnosis because Brucella spondylodiscitis may mimic spinal TB clinically and radiologically; however, systematic Brucella testing was not reported in this study (12). The study therefore cannot estimate the contribution of brucellosis.

Among confirmed TB cases with available susceptibility results, 13.8% had monoresistance and no multidrug resistance was documented. This finding differs from reports of higher drug resistance in some selected MDR spinal TB cohorts and should be interpreted in light of the small number tested and incomplete susceptibility data (6, 30).

### 4.4 Treatment and outcomes

Most patients were managed without an operative/procedural intervention, while 38.0% underwent an operative procedure. The most common operation was instrumentation with biopsy, reflecting the dual need for stabilization and tissue diagnosis in selected patients. Percutaneous biopsy was appropriately classified as a diagnostic procedure rather than a therapeutic intervention.

Among the 65 patients with documented follow-up, 70.8% achieved complete or partial recovery. However, almost half of the total cohort had no documented outcome data, creating potential attrition bias. Therefore, the 70.8% favorable-outcome proportion applies only to patients with documented follow-up and should not be generalized to the entire cohort. The absence of complete patient-level linkage for the lost-to-follow-up group also prevented a reliable comparison of baseline characteristics between those with and without documented follow-up.

Operative/procedural intervention was not significantly associated with outcome in the unadjusted comparison (Fisher's exact *p* = 0.399). Given the limited number of unfavorable outcomes and incomplete follow-up, no multivariable regression model was retained in the final manuscript. The outcome analysis is therefore descriptive and exploratory, and no causal inference can be made regarding operative/procedural intervention.

### 4.5 Interpretation of outcome associations

TB exposure history was associated with outcome in the bivariate analysis (*p* = 0.003). Trauma history was not statistically significant (*p* = 0.139), and operative/procedural intervention was not statistically significant (Fisher's exact *p* = 0.399). These unadjusted associations should be interpreted cautiously because the outcome analysis included only 65 patients and nearly half of the overall cohort had no documented follow-up.

Because outcome data were available for only 65 patients and 19 had unfavorable outcomes, the final analysis did not retain multivariable logistic regression. This decision avoids presenting unstable adjusted estimates from a sparse outcome dataset. The remaining bivariate findings are not adjusted for potential confounding and should not be interpreted as independent predictors or causal effects.

### 4.6 Strengths and limitations

The study provides local data from a tertiary referral hospital in Somalia and evaluates clinical, radiological, microbiological, treatment, and outcome variables within the same cohort. Use of a total-enumeration approach reduced sampling within the hospital-defined eligible population.

Several limitations should be considered. First, the retrospective single-center design limits generalizability and introduces potential selection and information bias. Second, microbiological or histopathological confirmation was available in only 38% of patients, limiting accurate determination of the etiological spectrum and creating potential misclassification, particularly because empirical anti-TB treatment was common. Third, systematic testing for Brucella and other alternative pathogens was not documented, so their contribution cannot be assessed. Fourth, outcome data were available for only 65 of 121 patients, introducing possible attrition bias. A patient-level comparison of baseline characteristics between the follow-up and lost-to-follow-up groups could not be reliably performed from the available record extract, so the direction and magnitude of attrition bias remain uncertain. Fifth, ASIA/Frankel grades were not consistently available or linked to outcomes at the patient level, preventing granular neurological recovery analysis. Sixth, small subgroups, including the high socioeconomic category, limited statistical precision. Finally, because no adjusted regression model was retained, the outcome comparisons are unadjusted and may be affected by confounding.

## 5 Conclusion

In this tertiary-care cohort from Somalia, spondylodiscitis commonly presented with back and radicular pain and was frequently accompanied by neurological deficit, spinal deformity, and epidural abscess. Confirmed TB represented 24.8% of the overall cohort and 65.2% of patients who underwent microbiological or histopathological testing, while 70.2% received empirical anti-TB treatment. Among patients with documented follow-up, 70.8% had favorable outcomes; this outcome estimate should not be generalized to the full cohort because 46.3% lacked documented follow-up. Neurological assessment was limited by incomplete ASIA/Frankel documentation. Outcome comparisons were unadjusted because the number of unfavorable outcomes and the extent of missing follow-up did not support reliable multivariable modeling. Improved microbiological diagnosis, consideration of alternative infections such as brucellosis, systematic neurological grading, and prospective multicenter follow-up are needed to better define the etiological spectrum and outcomes of spondylodiscitis in Somalia.

## Data Availability

The original contributions presented in the study are included in the article/Supplementary Material, further inquiries can be directed to the corresponding author.
